# People prefer joint outcome prosocial resource distribution towards future others

**DOI:** 10.1038/s41598-021-84796-4

**Published:** 2021-03-08

**Authors:** Yukako Inoue, Toshiyuki Himichi, Nobuhiro Mifune, Tatsuyoshi Saijo

**Affiliations:** 1 Research Institute for Future Design, Kochi University of Technology, Kochi, Japan; 2 School of Economics and Management, Kochi University of Technology, 2-22, Eikokuji-cho, Kochi-shi, Kochi 780-8515 Japan; 3 Research Institute for Humanity and Nature, Kyoto, Japan

**Keywords:** Psychology, Human behaviour

## Abstract

Today, developing and maintaining sustainable societies is becoming a notable social concern, and studies on altruism and prosociality toward future generations are increasing in importance. Although altruistic behaviors toward future generations have previously been observed in some experimental situations, it remains unknown whether prosocial preferences toward future others are based on equality or joint outcome orientations. In the present research, we exploratorily investigated preferences regarding resource distribution by manipulating the time points (i.e., present/future) of the participants and their imaginary partners. The results indicate that prosocial preference toward future others was as strong as that toward present others and seemed to be based on a joint outcome prosocial preference. Notably, when participants and their partners were at different time points, participants preferred to leave resources for the persons in the future. The findings indicate that the type of altruistic preference toward future others may differ from that toward present others, which is mainly equality.

## Introduction

In recent years, the development of sustainable societies (which is considered necessary for mitigating issues related to climate change and damage to the ecosystem) has become a major social concern^1^. However, when seeking to address these problems, prioritizing the benefit of the current generation can result in a deficit to the prospects of future generations. For example, the energy produced by fossil fuels enriches the lives of many people today, but the carbon dioxide emitted by such fuels can exacerbate climate change and lead to severe environmental problems in the future. These conflicts between the benefits of the present and future generations are referred to as “intergenerational sustainability dilemmas”^2^. To build sustainable societies, intergenerational sustainability dilemmas must be resolved; the present generation must act altruistically toward future generations—that is, restrict contemporary gains and instead emphasize the benefit of future generations. In accordance with the rising importance of this issue, the number of studies regarding decisions relating to intergenerational sustainability dilemmas has recently increased (e.g.,^2–6^). This study aims to detect and identify the type of altruistic preference for future generations related to intergenerational dilemmas.

Theoretically, it is difficult for the present generation to behave altruistically for the benefit of future generations in intergenerational sustainability dilemmas. This is because future generations cannot influence the decisions of the present generation. In other words, future generations cannot negotiate with the present generation even though the present generation’s decisions have important repercussions for them^7^. More specifically, considering that communication is known to promote altruistic behaviors in the context of social dilemmas^8^, it is likely that the lack of communication in intergenerational sustainability dilemmas constrains altruistic behavior toward future generations. Moreover, future generations cannot reciprocate with the present generation because the present generation leaves the social exchange context over time^9^. This is important to note because it is widely known that even self-interested individuals can display altruistic behavior toward others if there is a likelihood of reciprocity^10^. This reciprocity is not possible between future generations and the present generation; therefore, self-interested individuals are unmotivated to exhibit altruistic behaviors toward future generations.

Despite these difficulties, however, experimental research has revealed that people can be motivated to act altruistically toward future generations in intergenerational dilemmas. Although some studies that feature economic games indicate that most people do not choose the sustainable option in normal conditions, people have been found to behave altruistically toward subsequent generations when appropriate conditions are applied^11^. Moreover, studies that feature public goods games with intergenerational structures report that the majority of participants left sufficient resources for the next generation^3,6^. These studies demonstrate that people may have altruistic preferences for future generations. This consequently raises the question: What motivates us to exhibit altruistic behavior toward future generations?

Some studies address this question and demonstrate the psychological motivation for altruistic behavior to future others. One possible psychological theory explaining altruistic behavior toward future generations is the construal level theory^12^. This theory proposes that psychologically (e.g., temporally, spatially, and socially) distant objects are mentally represented abstractly, whereas psychologically close objects are mentally represented concretely. A meta-analysis study revealed a medium effect size of psychological distance on abstract thinking^13^. As the future is a more psychologically distant concept than the present, future thinking tends to lead to abstract thoughts. Therefore, when people focus on the future, they tend to make decisions based on abstract purposes, such as moral principles, more than those based on concrete interests or hedonistic values^14,15^. This can lead people to make more altruistic decisions. Studies on social discounting—in which people discount others’ resource values compared to their own resources—indicate that the discount rate becomes smaller in the distant future^16,17^. Although this is consistent with the prediction from the construal level theory, it is unclear what type of altruistic preference is facilitated by future thinking (i.e., temporal distance).

In addition, Wade-Benzoni and her colleagues suggested other psychological motivations for intergenerational altruistic preferences. For example, legacy motivation, which is the desire to leave something enduring and meaningful after one’s death, is an important factor^9^. Moreover, additional psychological factors increase legacy motivation and consequently promote altruistic behavior toward future others: affinity with future generations^18^, feeling of intergenerational reciprocity^19^, and sense of stewardship or social responsibility derived from extreme power asymmetry^20,21^. These psychological factors are known to motivate altruistic behaviors. However, what type of altruistic preference people have toward future others remains unknown. Therefore, in the current study, we examined the type of altruistic preference people have toward future others, such as whether people prefer to increase the sum of their own and future others’ benefits, or whether they prefer to have equal benefits for both themselves and future others.

To identify the type of altruistic preference for future others, we focused on social value orientation (SVO), which represents preferences related to resource distribution between the self and the other. A person’s SVO can theoretically be classified into several types^22,23^. However, most people are classified into one of three types: competitor, individualist, or prosocial^24,25^. The former two types are considered selfish preferences. Competitors prefer to maximize their gain relative to that of the other person, whereas individualists prefer to maximize their absolute gain, disregarding the other person’s outcome. In contrast to these two preferences, which are collectively called “proself” orientations, prosocials are cooperative or altruistic; they prefer maximizing the sum of their gain and their partners’ gain (this is also known as a “joint outcome” prosocial orientation, and is the same as the utilitarian concept in economics^26^). In addition, some studies have identified another type of prosocial orientation—the “equal outcome” prosocial; equal outcome prosocials prefer to minimize the difference between their gain and that of their partners^27^. Eek and Gärling^28^ revealed that most prosocial people prefer equal outcomes over joint outcomes. In other words, when distributing a resource among contemporaries, equality is the most prominent basis underlying prosocial behavior^29^.

People who have a prosocial preference in SVO tend to act more altruistically than those with either of the other two preferences^30,31^. Prosocials tend to show more cooperation in prisoners’ and public goods dilemma games^32,33^, fair allocation in dictator games^34^, and trusting and reciprocating behaviors in trust games^35,36^. Some of these types of behaviors have a latent factor of cooperativeness or altruism that correlates with SVO^36^. In addition to these experimental game studies, which were conducted in laboratories, prosocials exhibit altruistic behavior in real-life situations^37^. For example, prosocials prefer to use public transportation over private cars when commuting^38,39^, show a relatively high tendency to economize their water consumption^40^, and show strong pro-environmental intentions^41^. These findings indicate that prosocial preferences toward contemporaries measured by SVO affect not only altruistic behaviors toward contemporaries in real-life society but also the conservation of resources for the future, which directly relates to sustainability issues such as environmental problems. However, the above studies did not measure people’s preferences regarding future generations (or “future others”). Further, along with the fact that no previous study has considered prosocial preferences regarding future others, few studies have examined whether prosocial preferences regarding contemporaries affect their altruistic behavior toward future others. Timilsina et al.^42^ revealed that generativity, which relates to concern and commitment for the next generation, is positively associated with prosocial preference in SVO. Their study findings suggest that one’s SVO (i.e., their altruistic preference toward their contemporaries) affects his/her altruistic behavior toward future others; however, altruistic preference toward future others was not explicitly measured in their study. Furthermore, whether the altruistic preference toward future generations is based on joint outcomes, equal outcomes, or both remains unknown.

In the present study, our aim is not to investigate the psychological motivation for exhibiting altruistic behaviors toward future others; rather, our aim is to identify the type of altruistic preferences toward future others, if it exists. Therefore, we conducted a study using a modified version of the triple dominance measure of SVO^28^ to reveal whether joint outcomes and/or equal outcomes exist as altruistic preferences toward future generations. Furthermore, we exploratively investigated the effect that respective generations of decision-makers and their partners have on preferences regarding the distribution of resources. Concretely, we manipulated the generation (i.e., present/future) of the participant in addition to that of their imaginary counterpart and compared the proportions of each SVO type.

## Methods

### Participants

Overall, 824 participants affiliated with a research company (Macromill Inc., Japan) responded to our survey (329 men, 495 women; mean age = 39.57 years, standard deviation = 5.76, range: 30–49 years). We assigned over 200 participants per condition, which is only 38 less than the sample size required for detecting a stable correlation coefficient under typical effect sizes in personality and social psychology (*n* = 238)^43^.

We used the extended social value orientation task questionnaire^28^, adding the temporal difference of the self and other to the original definition of SVO. As we investigated the distribution between the self and future others in a way compatible with distribution in the present, we required participants to imagine a future time when they would likely still be alive. We chose to ask participants (in some conditions) to imagine themselves 40 years in the future. We consequently restricted our participants to adults in their 30 s and 40 s, who are more likely (compared to older individuals) to be alive in 40 years. Additionally, the task concerned the distribution of a valuable resource to future generations so we investigated whether parenthood influences this distribution. Japan has a low number of young adult parents due to the high proportion of unmarried adults (early 20 s: 93.2%; latter 20 s: 67.1%; Statistics Bureau of Japan, 2015^44^). Therefore, to investigate the effects of parenthood under sufficient dispersion, we excluded adults in their 20 s. Nearly half the participants in the present study were parents (not a parent: *n* = 403; parent: *n* = 421). The ethical committee of Kochi University of Technology approved this study’s procedures, which met the requirements of the Declaration of Helsinki. We obtained informed consent from all participants: we presented information regarding the study on the first page of the survey, and if the participants consented, they responded to the survey.

### Extended social value orientation task

Participants were presented with a matrix representing various distributions of fictional points between “the self” (the participant) and “the other” (a fictional player) and were asked to choose the most attractive option. The extended SVO task^28^ features four distribution types: individualistic, competitive, equality, and joint outcome. The individualistic distribution represents prioritizing the self’s benefit (e.g., 350 points to the self, 200 to the other). The competitive distribution represents maximizing the self’s relative benefit compared to that of the other (e.g., 300 to the self, 50 to the other). The equality distribution represents the self and the other getting the same points (e.g., 300 to the self, 300 to the other). The joint outcome distribution represents maximizing the sum of both points (e.g., 300 points to the self, 400 to the other). In this task, this distribution also represents self-sacrificing personal benefit in favor of that of the other.

**Figure 1:**
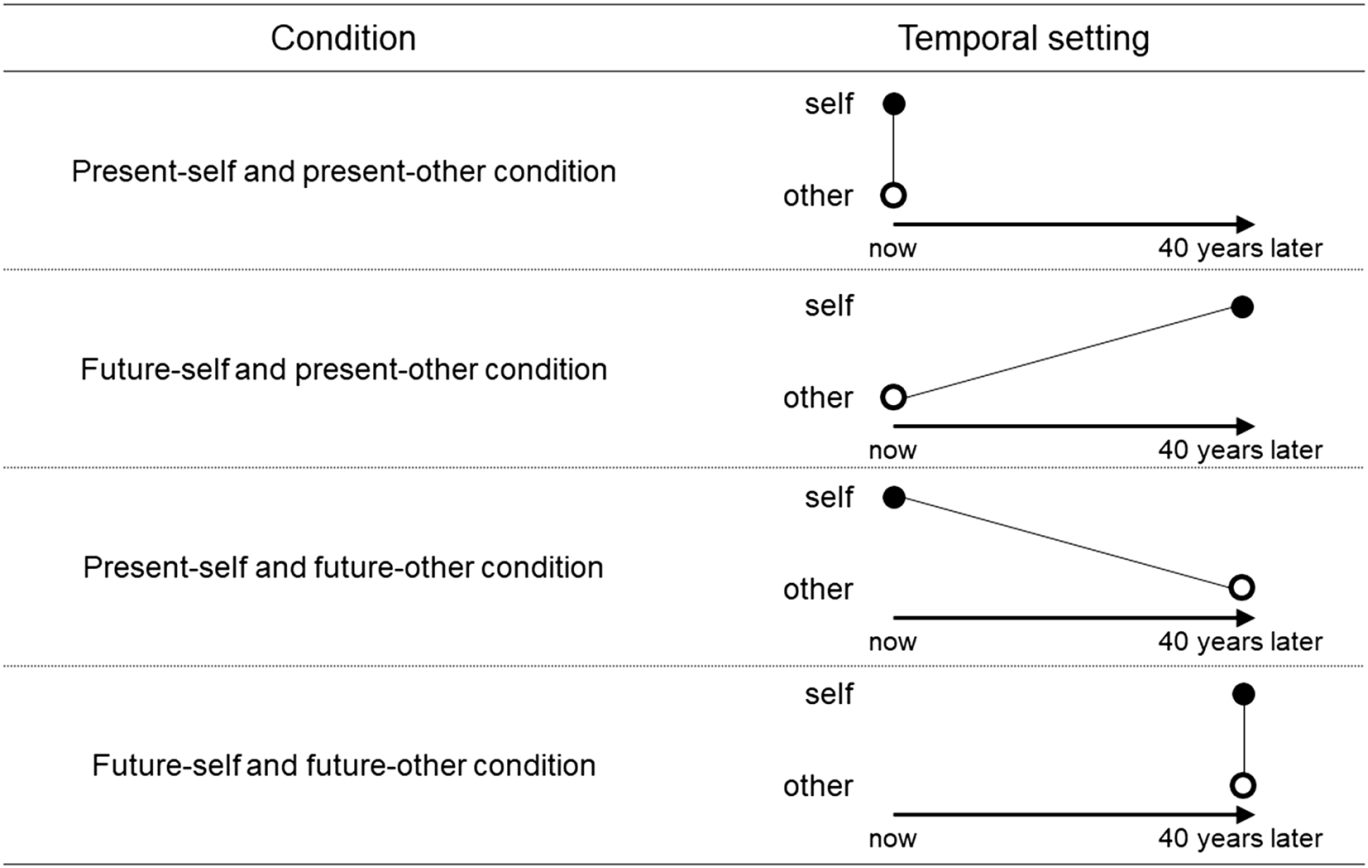
The temporal difference between self and other in each condition. Black dots represent self, and white dots represent other.

The basic task instructions and distribution matrices were based on previous studies^28,45^ (see Supplementary Appendix S1 and S2). We independently manipulated the time periods (i.e., present/future) of the two players—the self and the other. The task featured four conditions (present-self and present-other, future-self and present-other, present-self and future-other, and future-self and future-other). Participants were randomly assigned to a condition. In the present-self and present-other condition, participants imagined performing the task with a randomly selected stranger. In the future-self and present-other condition, participants imagined that they were 40 years in the future and 40 years older, and were performing the task with a randomly selected stranger from the present time. In the present-self and future-other condition, participants imagined performing the task with a randomly selected stranger living 40 years in the future. In the future-self and future-other condition, participants imagined that they were 40 years in the future and 40 years older, and were performing the task with a randomly selected stranger living 40 years in the future (Fig. 1). Participants responded to 12 matrices in total (the matrices’ presentation order was randomized across participants). Those who chose a particular distribution at least eight times (≧ 66.67%) were allocated to the corresponding SVO type^28^. Participants who did not choose any distribution at least eight times were categorized as having no SVO type.

Before the task, we asked 3 questions to confirm that the participants understood the instructions: “You should perform the task while imagining that you are paired with another person” (correct answer: “yes”); “While performing the task, you should not consider the points as valuable” (correct answer: “no”); and “Your decisions impact only the number of points that are distributed to you” (correct answer: “no”). Participants who incorrectly answered any question were excluded. The final sample size for each condition was 206, and the differences between the conditions regarding mean age and distribution of gender and parenthood were not significant (age: *F* (3, 820) = 0.66, *p* = 0.579, η_p_^2^ < 0.01; gender: χ^2^ (3) = 2.81, *p* = 0.422; parenthood: χ^2^ (3) = 1.49, *p* = 0.684; details in Supplemental Appendix S4).

### Procedure

This study was conducted online. Participants initially responded to the extended SVO task and then to five personality scales that could be related to altruism toward future others. We measured these personality scales only for exploratory purposes, but we determined their inclusion in the main text was inappropriate, as their analyses differed from the main purpose of this study. Instead, the scales and results are described in Supplemental Appendix S3–S5 from the viewpoint of data publication. The response orders for the scales and the items in each scale were randomized. There were no instances of missing data because the research company’s system required responses for all items. The participants were permitted to cease answering the survey at any time.

### Data analysis

The descriptive statistics were calculated using the psych package^46^ (ver. 1.9.12) for R^47^ (ver. 3.6.2), and a one-way between-participant analysis of variance (ANOVA) was conducted using Anovakun^48^ (ver. 4.8.2) for R. Additionally, we tested for differences in the distribution of SVO type among conditions using chi-square tests. If these tests produced significant results, a residual analysis was conducted to determine which cell differed significantly from the expected value. We corrected the *p* value of this test using Bonferroni’s method. Thirty-eight participants could not be categorized into any SVO type (present-self and present-other: *n* = 7; future-self and present-other: *n* = 14; present-self and future-other: *n* = 9; future-self and future-other: *n* = 8) and were excluded from the chi-square analysis. Additionally, because only a few participants were categorized as competitive (present-self and present-other: *n* = 3; future-self and present-other: *n* = 6; present-self and future-other: *n* = 0; future-self and future-other: *n* = 3), we combined competitive with individualistic to create “proself” in accordance with previous methodologies^28,45^. Therefore, a 3 (SVO) × 4 (condition) cross table was created. We set the significance level of all statistical tests at 0.05 (two-tailed).

We also conducted hierarchical logistic regression analysis using R. In step 1, we entered age and a dummy variable for gender (1 = men, 2 = women) and parenthood (1 = not a parent, 2 = parent) as control variables, and dummy variables for each SVO type were entered as dependent variables (dummy proself: 0 = none, equality, and joint outcome, 1 = competitive and individualistic; dummy equality: 0 = none, competitive, individualistic, and joint outcome, 1 = equality; dummy joint outcome: 0 = none, competitive, individualistic, and equality, 1 = joint outcome), respectively. In step 2, we added dummy variables for the self and the other conditions (both: 0 = present, 1 = future) and their interaction as prediction variables. All prediction variables were mean-centered. Cox and Snell R-squares and Akaike’s information criteria (AIC) for each step were calculated using the PseudoR2 package^49^ (ver. 0.5) for R. Model comparison was conducted using the ANOVA function. If interaction effects were significant, we conducted a simple slope analysis. We also compared a model that included control variables (age and dummy variable for gender and parenthood) and the prediction variable to a model that included only control variables.

## Results

### Chi-square analysis

Chi-square analysis for the 3 (SVO) × 4 (condition) cross table demonstrated significance (Table 1: χ^2^(6) = 71.29, *p* < 0.001) and we conducted a residual analysis. In the present-self and present-other condition, compared to the expected value, the frequency of equality was significantly higher (Bonferroni-corrected *p* < 0.001), and the frequency of joint outcome was significantly lower (Bonferroni-corrected *p* < 0.001). In the future-self and present-other condition, compared to the expected value, the frequency of proself was significantly higher (Bonferroni-corrected *p* = 0.006), and the frequency of equality was significantly lower (Bonferroni-corrected *p* < 0.001). In the present-self and future-other condition, compared to the expected value, the frequency of joint outcome was significantly higher (Bonferroni-corrected *p* < 0.001), and the frequency of equality was significantly lower (Bonferroni-corrected *p* = 0.035). In the future-self and future-other condition, the frequencies of all SVO categories did not significantly differ from the respective expected values (Bonferroni-corrected *p*s > 0.05).

**Table 1:** The number of participants with each social value orientation in each condition.

	Proself		Equality		Joint outcome	
Present/Present	24	(3.05%)	149***	(18.96%)	26***	(3.31%)
Future/Present	42**	(5.34%)	76***	(9.67%)	74	(9.41%)
Present/Future	22	(2.80%)	88*	(11.20%)	87***	(11.07%)
Future/Future	24	(3.05%)	110	(13.99%)	64	(8.14%)

***: Bonferroni-corrected *p* < .001; **: Bonferroni-corrected *p* < .010; *: Bonferroni-corrected *p* < .050.

### Logistic analysis

For the dummy variable for proself, the step-2 model showed a significantly better goodness of fit than did the step-1 model (Table 2; ΔAIC =  − 4.13, ΔR^2^ = 0.01, *p* = 0.017). However, the effects of the dummy variables of the self and the other and their interaction were not significant in the step-2 model (0.55 < odds ratios [*OR*s] < 1.44; *p* > 0.070).

**Table 2:** Results of logistic regressions for the proself dummy variable.

DV = dummy proself	Step 1					Step 2				
(0: none, equality, joint outcome; 1: competitive, individualistic)	Coef	*SE*	*Z*	*p*	*OR*	Coef	*SE*	*Z*	*p*	*OR*
Gender (1: men; 2: women)	− 0.22	0.21	− 1.02	.308	0.80	− 0.20	0.21	− 0.93	.353	0.82
Age	− 0.00	0.02	− 0.02	.982	1.00	0.00	0.02	0.07	.944	1.00
Parenthood (1: not a parent, 2: parent)	− 0.31	0.21	− 1.47	.141	0.74	− 0.32	0.21	− 1.52	.129	0.73
Dummy_self (0: present; 1: future)						0.37	0.21	1.75	.080	1.44
Dummy_other (0: present; 1: future)						− 0.38	0.21	− 1.81	.070	0.68
Dummy_self × dummy_other						− 0.60	0.42	− 1.44	.150	0.55
Model fit	AIC = 659.19, R^2^ < .01					AIC = 655.06, R^2^ = .02				
						ΔAIC = − 4.13, ΔR^2^ = .01, *p* = .017				

R^2^ indicates Cox and Snell R^2^ values.

Coef. = coefficient; SE = standard error; OR = odds ratio; AIC = Akaike’s information criteria.

**Figure 2:**
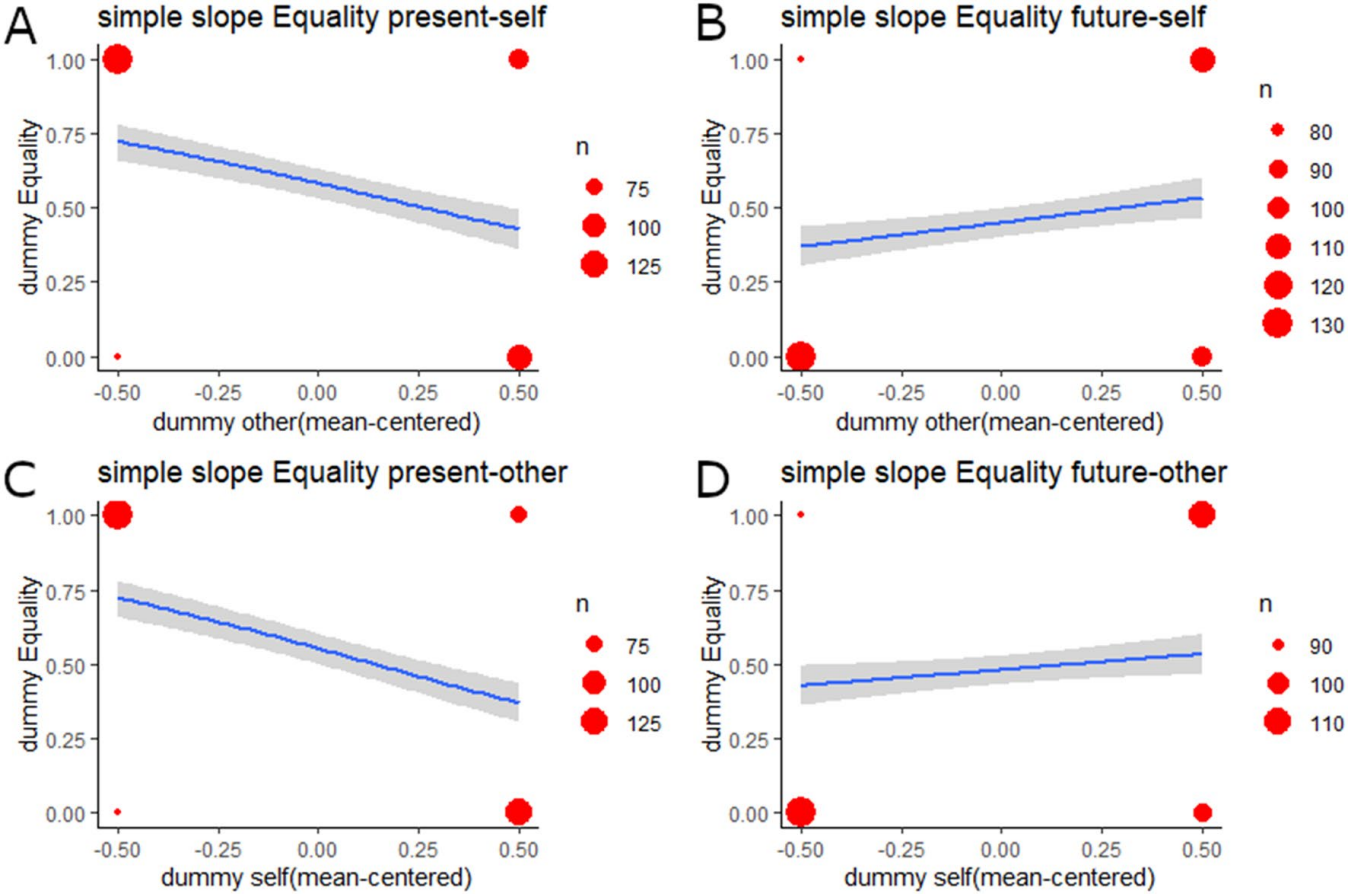
Results of simple slope analyses for Equality preference. Size of the red dots indicates sample size. Blue lines are regression lines, and gray-colored areas indicate 95% confidence intervals. (**A**) Simple effects of other condition in the present-self condition (self dummy = 0). (**B**) Simple effects of other condition in the future-self condition (self dummy = 1). (**C**) Simple effects of self condition in the present-other condition (other dummy = 0). (**D**) Simple effects of self condition in the future-other condition (other dummy = 1). This figure was drawn using the ggplot2 package^53^ (ver. 3.2.0) for R.

For the dummy variable for equality, the step-2 model also had a significantly better goodness of fit than did the step-1 model (Table 3; ΔAIC =  − 58.23, ΔR^2^ = 0.07, *p* < 0.001). In the step-2 model, the effects of the dummy variables for the self and the other were negative and significant (self: *OR* = 0.61, *p* = 0.001; other: *OR* = 0.73, *p* = 0.037). More importantly, the interaction effect was also significant (*OR* = 7.96, *p* < 0.001). In the simple slope analysis of all models, the step-2 model showed a significantly better fit than did the step-1 model (Supplementary Table S1–S4; ΔAICs <  − 5.13, ΔR^2^s > 0.02, *p* < 0.008). In the sub-sample featuring the present-self condition (for which the dummy variable for the self = 0), the simple effect of the dummy variable was negative and significant (Fig. 2A and Supplementary Table S1; *OR* = 0.24, *p* < 0.001). In the sub-sample featuring the future-self condition (dummy variable for the self = 1), the simple effect of the dummy variable was positive and significant (Fig. 2B and Supplementary Table S2; *OR* = 2.02, *p* = 0.001). In the sub-sample featuring the present-other condition (dummy variable for the other = 0), the simple dummy variable for the self was affected negatively and significantly (Fig. 2C and Supplementary Table S3; *OR* = 0.22, *p* < 0.001). In the sub-sample featuring the future-other condition (dummy variable for the other = 1), the dummy variable for the self was affected positively and significantly (Fig. 2D and Supplementary Table S4; *OR* = 1.74, *p* = 0.008).

**Table 3:** Results of logistic regressions for the equality dummy variable.

DV = dummy equality	Step 1					Step 2				
(0: none, competitive, individualistic, joint outcome; 1: equality)	Coef	*SE*	*Z*	*p*	*OR*	Coef	*SE*	*Z*	*p*	*OR*
Gender (1: men; 2: women)	0.87	0.15	5.74	< .001	2.40	0.95	0.16	5.92	< .001	2.58
Age	0.03	0.01	2.16	.031	1.03	0.03	0.01	1.92	.054	1.03
Parenthood (1: not a parent, 2: parent)	− 0.01	0.14	− 0.09	.927	0.99	0.02	0.15	0.11	.912	1.02
Dummy_self (0: present; 1: future)						− 0.50	0.15	− 3.37	.001	0.61
Dummy_other (0: present; 1: future)						− 0.31	0.15	− 2.09	.037	0.73
Dummy_self × dummy_other						2.07	0.30	6.90	< .001	7.96
Model fit	AIC = 1,114.18, R^2^ = .04					AIC = 1,055.95, R^2^ = .11				
						ΔAIC = − 58.23, ΔR^2^ = .07, *p* < .001				

R^2^ indicates Cox and Snell R^2^ values.

Coef. = coefficient; SE = standard error; OR = odds ratio; AIC = Akaike’s information criteria.

**Figure 3:**
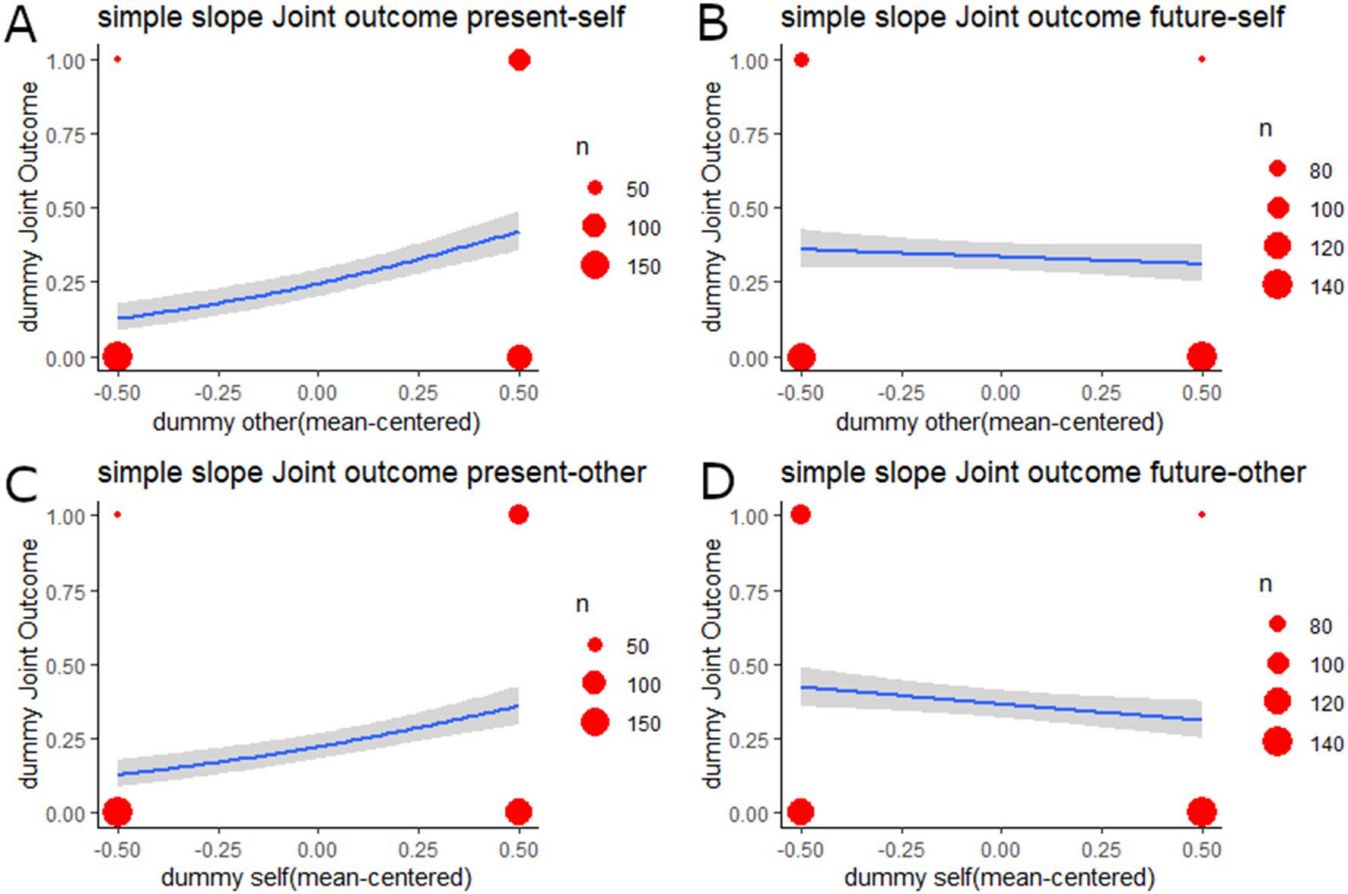
Results of simple slope analyses for Joint outcome preference. Size of the red dots indicates sample size. Blue lines are regression lines, and gray-colored areas indicate 95% confidence intervals. (**A**) Simple effects of other condition in the present-self condition (self dummy = 0). (**B**) Simple effects of other condition in the future-self condition (self dummy = 1). (**C**) Simple effects of self condition in the present-other condition (other dummy = 0). (**D**) Simple effects of self condition in the future-other condition (other dummy = 1). This figure was drawn using the ggplot2 package^53^ (ver. 3.2.0) for R.

For the dummy variable for joint outcome, the step-2 model showed a significantly better goodness of fit than did the step-1 model (Table 4; ΔAIC =  − 49.29, ΔR^2^ = 0.06, *p* < 0.001). In the step-2 model, the effects on the dummy variables for the self and the other were positive and significant (*OR*s > 1.48, *p* < 0.020), and their interaction was negative and significant (*OR* = 0.14, *p* < 0.001). In the simple slope analysis models, goodness of fit was higher in the step-2 model than in the step-1 model for three models (Supplementary Tables S5, S7, and S8; ΔAICs <  − 6.08, ΔR^2^s > 0.02, *p* < 0.004), but was not significantly higher in one model (Supplementary Table S6; ΔAIC = 0.77, ΔR^2^ < 0.01, *p* = 0.268). In the sub-sample featuring the present-self condition (dummy variable for the self = 0), the effect on the dummy variable for the other was positive and significant (Fig. 3A and Supplementary Table S5; *OR* = 6.51, *p* < 0.001), whereas in the sub-sample featuring the future-self condition (dummy variable for the self = 1), such an effect was not significant (Fig. 3B and Supplementary Table S6; *OR* = 0.79, *p* = 0.269). In the sub-sample featuring the present-other condition (Fig. 3C and dummy variable for the other = 0), the dummy variable for the self was affected positively and significantly (Supplementary Table S7; *OR* = 3.93, *p* < 0.001), whereas it was negatively and significantly affected in the sub-sample featuring the future-other condition (dummy variable for the other = 1; Fig. 3D and Supplementary Table S8; *OR* = 0.54, *p* = 0.005).

**Table 4:** Results of logistic regressions for the joint outcome dummy variable.

DV = dummy joint outcome	Step 1					Step 2				
(0: none, competitive, individualistic, equality, 1: joint outcome)	Coef	*SE*	*Z*	*p*	*OR*	Coef	*SE*	*Z*	*p*	*OR*
Gender (1: men; 2: women)	− 1.00	0.16	− 6.14	< .001	0.37	− 1.08	0.17	− 6.36	< .001	0.34
Age	− 0.02	0.01	− 1.69	.091	0.98	− 0.02	0.01	− 1.44	.151	0.98
Parenthood (1: not a parent, 2: parent)	0.13	0.16	0.80	.426	1.13	0.11	0.16	0.66	.507	1.11
Dummy_self (0: present; 1: future)						0.39	0.17	2.33	.020	1.48
Dummy_other (0: present; 1: future)						0.74	0.17	4.41	< .001	2.10
Dummy_self × dummy_other						− 1.99	0.34	− 5.87	< .001	0.14
Model fit	AIC = 982.21, R^2^ = .05					AIC = 932.92, R^2^ = .11				
						ΔAIC = − 49.29, ΔR^2^ = .06, *p* < .001				

R^2^ indicates Cox and Snell R^2^ values.

Coef. = coefficient; SE = standard error; OR = odds ratio; AIC = Akaike’s information criteria.

## Discussion

The present study’s findings suggest that a prosocial preference toward future others exists. Although intertemporal distance may increase psychological distance and inhibit prosocial behaviors^18^, the number of participants in our experiment who chose prosocial distribution in the present-self and future-other condition was the same as the number that chose this distribution type in the present-self and present-other condition. This indicates that people have a prosocial preference toward future others, similar to, if not stronger than, that toward present others. However, the type of these prosocial preferences may differ. As reported by Eek and Gärling^28^, our participants tended to prefer equality in the present-self and present-other condition; contrary to this, they tended to prefer joint outcome distribution in the present-self and future-other condition. This suggests that prosocial preference toward future others is based on joint outcome distribution, unlike that for present others. Thus, it is likely that prosocial preferences toward present others and future others have different psychological bases.

Moreover, the significant interaction effect of the self and the other conditions in the logistic regression indicated that differences in the generation between decision-makers and their partners affect distribution preferences. Regarding equality preference, simple slope effects were negative when the self or the other was in the present condition. In other words, the equality was preferred less in the future-other condition than in the present-other condition when the self was in the present condition, and it was also preferred less in the future-self condition than in the present-self condition when the other was in the present condition. In contrast, these simple slope effects were positive when the self or the other was in the future condition. This demonstrates that equality was preferred more in the future-other condition than in the present-other condition when the self was in the future condition, and it was also preferred more in the future-self condition than in the present-self condition when the other was in the future condition. These results indicate that people tend to prefer equal distribution when they are interacting with partners who are at the same time point. Regarding joint outcome preference, simple slope effects were positive when the self or the other was in the present condition, whereas these effects were negative when the other was in the future condition. The former indicates that the joint outcome was preferred more in the future-other condition than in the present-other condition when the self was in the present condition and more in the future-self condition than in the present-self condition when the other was in the present condition. The latter demonstrates that joint outcome was preferred less in the future-self condition than in the present-self condition when the other was in the future condition. Therefore, when decision-makers and their partners are at different time points, people tend to prefer leaving more resources to the person who is in the future. This implies that, in addition to a prosocial preference toward present others, people possess a form of future-orientation; that is, a tendency to conserve more resources for the future. The significantly higher frequency of proself in the future-self and present-other condition may also support this.

In the psychological field, some social-discounting-focused studies have investigated transitions in the subjective value of others’ resources in the future^16^. Osiński and Karbowski^17^ revealed that the relative value of the resources of socially distant others slightly increases in the future. This is consistent with our results. However, these studies considered situations in which the resources could not be shared between the participants and their partners (i.e., one party obtains all resources); our study investigated resource distribution and revealed distribution preferences. Moreover, the result when oneself and one’s partner were at different time points is also an original finding of our study.

Although the present study findings suggest that people have prosocial preferences toward future others, as mentioned above, in real-life situations most people seem to prioritize the present generation’s benefit. Some experimental studies have also indicated that people seldom behave altruistically toward future generations in normal conditions (e.g.,^2,11^). What creates this difference? The first possibility is that consideration for present others prevents prosocial behavior in the future. Even if people have prosocial preferences toward others, they may refrain from exhibiting prosocial behaviors that reduce the present generation’s benefit as a result of a fear of criticism from present others. In contrast, in this study, the participants could make decisions without concern about the opinions of present others as only two persons (the self and the other) were involved in the resource distribution. This may explain why our participants tended to behave more prosocially when compared to other experimental studies in which others also participated in the interaction^2,50^. The second possibility is that people tend to be more likely to engage in prosocial behavior toward a specific future individual than to anonymous future people. This effect has previously been observed in prosocial behavior toward present others. For example, Small and Loewenstein^51^ indicated that prosocial behavior toward an identified individual is promoted when compared to that toward an anonymous person; moreover, Kogut and Ritov^52^ revealed that this effect can only be observed in the prosocial behavior of a single individual, not a group. If this effect is also present in the context of prosocial behavior toward future generations, our participants may have tended to act more prosocially in this study because they were asked to imagine a specific individual as their partner. Another possibility is that social desirability bias affected the participants’ responses. In our study, the number of participants allocated to the proself type was small when compared to the number reported in Eek and Gärling^28^. This may be because socially desirable preference rather than prosocial preference was measured. There are several possible causes of social desirability bias in our experiment; for example, the fact that the participants’ decisions did not incur any benefits or costs, and that participants may have found it difficult to imagine an interaction with their partner 40 years in the future. Future studies should measure prosocial preferences while excluding these possibilities.

In the present study, we did not ask participants whether they believed they would be alive in forty years. Resource allocation for future others might be influenced by the expectation of remaining alive. If the participants believed that they would not be alive forty years later, legacy motivation^9^ may have facilitated the prosocial motivation for future others. Future studies should investigate the influence of cognition of life span on prosocial behavior for future others.

Despite these limitations, this study produced meaningful findings, as it identified the distribution preference toward (hypothetical) future generations. This differs from most previous related experimental research, in which contemporary participants represent different generations for convenience^2,3^. As this research represents the first attempt at such an investigation, some additional elements must be examined in future research. First, this study indicated that people may have a preference to leave more resources for the future than to implement equal distribution. However, it remains unknown whether this preference was observed because the participants earned the same amount of benefit by choosing the joint outcome as when choosing the equality option, or whether the participants were displaying an altruistic preference, and would have been willing to decrease their resources further to give future people more. Altruism, in which one prefers to maximize their partner’s absolute gain while disregarding the outcome of himself/herself, can be theoretically considered an SVO type^22^; however, people who prefer to give resources to present others, even if their resources are less than the equality, are so rare that we did not add this choice. Nevertheless, some people may have an altruistic preference regarding resource distribution with future others. This is a topic for future discussion. Next, this study investigated prosocial preferences using a point in the future when the participants were likely to still be alive. This preference might be different for more distant points in the future; that is, at points when the participants would be deceased. Therefore, future studies should investigate the changes in distribution preferences as “the future” time point changes. Finally, further research is also needed to reveal whether this preference can actually lead to altruistic behavior toward future others. In the current study, we focused on revealing the type of altruistic basis toward the future in comparison with SVO. Thereafter, we used an abstract resource distribution task with a questionnaire to measure SVO. As mentioned in the introduction, the prosocial preference of SVO predicts altruistic behaviors in real-life situations and sustainable behaviors in environmental problems. Therefore, the distribution preference in our experiment can predict realistic behaviors to a certain extent. However, this prediction should be confirmed by the distribution task in more realistic situations. Whether certain manipulations can increase altruism toward the future through joint outcome prosocial preference should also be investigated.

## Supplementary Information

Supplementary Information

## Data Availability

The datasets and R cords generated during and/or analyzed during the current study are available in the open science framework, https://osf.io/mt95j/?view_only=b29161ed1c304e7bbad274b5a485e16d.
