# Supplementary material for: People prefer joint outcome prosocial resource distribution towards future others
Source: Sci Rep. 2021 Mar 8;11:5373. doi: 10.1038/s41598-021-84796-4 (PMC7940491; doi:10.1038/s41598-021-84796-4)
Supplement: Supplementary file 1 — Supplementary Information [file 41598_2021_84796_MOESM1_ESM.doc]

Title page

**Title:** People prefer joint outcome prosocial resource distribution towards future others

Yukako Inoue^1^, Toshiyuki Himichi^1,2^, Nobuhiro Mifune^1,2^, and Tatsuyoshi Saijo^1,2,3^

^1^Research Institute for Future Design, Kochi University of Technology, Kochi, Japan

^2^School of Economics and Management, Kochi University of Technology, Kochi, Japan

^3^Research Institute for Humanity and Nature, Kyoto, Japan

**Abbreviations**

Coef. = coefficient

SE = standard error

OR = odds ratio

AIC = Akaike's information criteria

IRI: Interpersonal Reactivity Index.

CFC: Consideration of Future Consequences

GCS: Generative Concern Scale

TIPI: Ten Items Personality Inventory

PD: Personal Distress

EC: Empathic Concern

PT: Perspective Taking

FS: Fantasy

Ext.: Extraversion

Agr.: Agreeableness

Con: Conscientiousness

Neu.: Neuroticism

Ope.: Openness

Supplementary Table S1.

*Results of simple slope analysis of logistic regressions for dummy equality* (dummy self = 0).

| **DV = dummy Equality** | Step 1 |  |  |  |  |  | Step 2 |  |  |  |  |  |
| --- | --- | --- | --- | --- | --- | --- | --- | --- | --- | --- | --- | --- |
| (0: none, Competition, Individualistic, Joint outcome; 1: Equality) | Coef. | *SE* | *Z* | *p* | *OR* |  | Coef. | *SE* | *Z* | *p* | *OR* |  |
| gender (1: men; 2: women) | 1.14 | 0.22 | 5.11 | <.001 | 3.12 |  | 1.33 | 0.24 | 5.51 | <.001 | 3.77 |  |
| age | 0.03 | 0.02 | 1.83 | .068 | 1.03 |  | 0.03 | 0.02 | 1.70 | .089 | 1.03 |  |
| Parenthood (1: not a parent, 2: parent) | -0.08 | 0.21 | -0.36 | .719 | 0.93 |  | -0.04 | 0.22 | -0.18 | .861 | 0.96 | |
| dummy_other (0: present; 1: future) |  |  |  |  |  |  | -1.42 | 0.23 | -6.29 | <.001 | 0.24 |  |
| Model fit | AIC = 541.63, R^2^ = .07 | | | | |  | AIC = 500.48, R^2^ = .16 | | | | |  |
|  |  | | | | |  | ΔAIC = -41.15, ΔR^2^ = .09, *p* < .001 | | | | |  |

*Note*. R^2^ indicates Cox and Snell R^2^.

Supplementary Table S2.

*Results of simple slope analysis of logistic regressions for dummy equality* (dummy self = 1).

| **DV = dummy Equality** | Step 1 |  |  |  |  |  | Step 2 |  |  |  |  |
| --- | --- | --- | --- | --- | --- | --- | --- | --- | --- | --- | --- |
| (0: none, Competition, Individualistic, Joint outcome; 1: Equality) | Coef. | *SE* | *Z* | *p* | *OR* |  | Coef. | *SE* | *Z* | *p* | *OR* |
| gender (1: men; 2: women) | 0.60 | 0.21 | 2.81 | .005 | 1.81 |  | 0.62 | 0.22 | 2.89 | .004 | 1.87 |
| age | 0.02 | 0.02 | 1.01 | .313 | 1.02 |  | 0.02 | 0.02 | 1.03 | .304 | 1.02 |
| Parenthood (1: not a parent, 2: parent) | 0.03 | 0.20 | 0.13 | .897 | 1.03 |  | 0.05 | 0.21 | 0.26 | .792 | 1.06 |
| dummy_other (0: present; 1: future) |  |  |  |  |  |  | 0.70 | 0.20 | 3.45 | .001 | 2.02 |
| Model fit | AIC = 566.78, R^2^ = .02 | | | | |  | AIC = 556.68, R^2^ = .05 | | | | |
|  |  | | | | |  | ΔAIC = -10.10, ΔR^2^ = .03, *p* = .001 | | | | |

*Note*. R^2^ indicates Cox and Snell R^2^.

Supplementary Table S3.

*Results of simple slope analysis of logistic regressions for dummy equality* (dummy other = 0).

| **DV = dummy Equality** | Step 1 |  |  |  |  |  | Step 2 |  |  |  |  |
| --- | --- | --- | --- | --- | --- | --- | --- | --- | --- | --- | --- |
| (0: none, Competition, Individualistic, Joint outcome; 1: Equality) | Coef. | *SE* | *Z* | *p* | *OR* |  | Coef. | *SE* | *Z* | *p* | *OR* |
| gender (1: men; 2: women) | 0.66 | 0.22 | 3.03 | .002 | 1.93 |  | 0.69 | 0.23 | 2.97 | .003 | 1.99 |
| age | 0.04 | 0.02 | 1.97 | .049 | 1.04 |  | 0.03 | 0.02 | 1.58 | .114 | 1.03 |
| Parenthood (1: not a parent, 2: parent) | 0.19 | 0.20 | 0.92 | .359 | 1.21 |  | 0.25 | 0.22 | 1.15 | .249 | 1.29 |
| dummy_self (0: present; 1: future) |  |  |  |  |  |  | -1.52 | 0.22 | -7.01 | <.001 | 0.22 |
| Model fit | AIC = 562.46, R^2^ = .03 | | | | |  | AIC = 511.47, R^2^ = .15 | | | | |
|  |  | | | | |  | ΔAIC = -50.99, ΔR^2^ = .12, *p* < .001 | | | | |

*Note*. R^2^ indicates Cox and Snell R^2^.

Supplementary Table S4.

*Results of simple slope analysis of logistic regressions for dummy equality* (dummy other = 1).

| **DV = dummy Equality** | Step 1 |  |  |  |  |  | Step 2 |  |  |  |  |  |
| --- | --- | --- | --- | --- | --- | --- | --- | --- | --- | --- | --- | --- |
| (0: none, Competition, Individualistic, Joint outcome; 1: Equality) | Coef. | *SE* | *Z* | *p* | *OR* |  | Coef. | *SE* | *Z* | *p* | *OR* |  |
| gender (1: men; 2: women) | 1.12 | 0.22 | 5.10 | <.001 | 3.06 |  | 1.18 | 0.22 | 5.30 | <.001 | 3.26 |  |
| age | 0.02 | 0.02 | 0.99 | .324 | 1.02 |  | 0.02 | 0.02 | 1.09 | .275 | 1.02 |  |
| Parenthood (1: not a parent, 2: parent) | -0.21 | 0.21 | -1.01 | .311 | 0.81 |  | -0.19 | 0.21 | -0.89 | .372 | 0.83 | |
| dummy_self (0: present; 1: future) |  |  |  |  |  |  | 0.55 | 0.21 | 2.65 | .008 | 1.74 |  |
| Model fit | AIC = 551.09, R^2^ = .06 | | | | |  | AIC = 545.96, R^2^ = .08 | | | | |  |
|  |  | | | | |  | ΔAIC = -5.13, ΔR^2^ = .02, *p* = .008 | | | | |  |

*Note*. R^2^ indicates Cox and Snell R^2^.

Supplementary Table S5.

Results of simple slope analysis of logistic regressions for dummy joint outcome (dummy self = 0).

| **DV = dummy Joint outcome** | Step 1 |  |  |  |  |  | Step 2 |  |  |  |  |  |
| --- | --- | --- | --- | --- | --- | --- | --- | --- | --- | --- | --- | --- |
| (0: none, Equality, Competition, Individualistic; 1: Joint outcome) | Coef. | *SE* | *Z* | *p* | *OR* |  | Coef. | *SE* | *Z* | *p* | *OR* |  |
| gender (1: men; 2: women) | -1.40 | 0.25 | -5.69 | <.001 | 0.25 |  | -1.68 | 0.27 | -6.15 | <.001 | 0.19 |  |
| age | -0.04 | 0.02 | -1.79 | .074 | 0.96 |  | -0.03 | 0.02 | -1.51 | .132 | 0.97 |  |
| Parenthood (1: not a parent, 2: parent) | 0.23 | 0.24 | 0.97 | .333 | 1.26 |  | 0.21 | 0.26 | 0.83 | .407 | 1.24 | |
| dummy_other (0: present; 1: future) |  |  |  |  |  |  | 1.87 | 0.28 | 6.78 | <.001 | 6.51 |  |
| Model fit | AIC = 457.59, R^2^ = .08 | | | | |  | AIC = 404.58, R^2^ = .20 | | | | |  |
|  |  | | | | |  | ΔAIC = -53.01, ΔR^2^ = .11, *p* < .001 | | | | |  |

*Note*. R^2^ indicates Cox and Snell R^2^.

Supplementary Table S6.

Results of simple slope analysis of logistic regressions for dummy joint outcome (dummy self = 1).

| **DV = dummy Joint outcome** | Step 1 |  |  |  |  |  | Step 2 |  |  |  |  |  |
| --- | --- | --- | --- | --- | --- | --- | --- | --- | --- | --- | --- | --- |
| (0: none, Equality, Competition, Individualistic; 1: Joint outcome) | Coef. | *SE* | *Z* | *p* | *OR* |  | Coef. | *SE* | *Z* | *p* | *OR* |  |
| gender (1: men; 2: women) | -0.64 | 0.22 | -2.92 | .004 | 0.53 |  | -0.65 | 0.22 | -2.94 | .003 | 0.52 |  |
| age | -0.01 | 0.02 | -0.52 | .606 | 0.99 |  | -0.01 | 0.02 | -0.52 | .605 | 0.99 |  |
| Parenthood (1: not a parent, 2: parent) | 0.06 | 0.21 | 0.26 | .793 | 1.06 |  | 0.05 | 0.21 | 0.23 | .819 | 1.05 | |
| dummy_other (0: present; 1: future) |  |  |  |  |  |  | -0.23 | 0.21 | -1.11 | .269 | 0.79 |  |
| Model fit | AIC = 524.73, R^2^ = .02 | | | | |  | AIC = 525.50, R^2^ = .02 | | | | |  |
|  |  | | | | |  | ΔAIC = 0.77, ΔR^2^ < .01, *p* = .268 | | | | |  |

*Note*. R^2^ indicates Cox and Snell R^2^.

Supplementary Table S7.

Results of simple slope analysis of logistic regressions for dummy joint outcome (dummy other = 0).

| **DV = dummy Joint outcome** | Step 1 |  |  |  |  |  | Step 2 |  |  |  |  |
| --- | --- | --- | --- | --- | --- | --- | --- | --- | --- | --- | --- |
| (0: none, Equality, Competition, Individualistic; 1: Joint outcome) | Coef. | *SE* | *Z* | *p* | *OR* |  | Coef. | *SE* | *Z* | *p* | *OR* |
| gender (1: men; 2: women) | -0.89 | 0.25 | -3.56 | <.001 | 0.41 |  | -0.90 | 0.26 | -3.48 | .001 | 0.41 |
| age | -0.04 | 0.02 | -1.92 | .055 | 0.96 |  | -0.03 | 0.02 | -1.57 | .118 | 0.97 |
| Parenthood (1: not a parent, 2: parent) | -0.11 | 0.24 | -0.46 | .644 | 0.90 |  | -0.15 | 0.25 | -0.59 | .553 | 0.86 |
| dummy_self (0: present; 1: future) |  |  |  |  |  |  | 1.37 | 0.26 | 5.26 | <.001 | 3.93 |
| Model fit | AIC = 449.27, R^2^ = .04 | | | | |  | AIC = 420.61, R^2^ = .11 | | | | |
|  |  | | | | |  | ΔAIC = -28.66, ΔR^2^ = .07, *p* < .001 | | | | |

*Note*. R^2^ indicates Cox and Snell R^2^.

Supplementary Table S8.

Results of simple slope analysis of logistic regressions for dummy joint outcome (dummy other = 1).

| **DV = dummy Joint outcome** | Step 1 |  |  |  |  |  | Step 2 |  |  |  |  |
| --- | --- | --- | --- | --- | --- | --- | --- | --- | --- | --- | --- |
| (0: none, Equality, Competition, Individualistic; 1: Joint outcome) | Coef. | *SE* | *Z* | *p* | *OR* |  | Coef. | *SE* | *Z* | *p* | *OR* |
| gender (1: men; 2: women) | -1.15 | 0.22 | -5.19 | <.001 | 0.32 |  | -1.22 | 0.23 | -5.40 | <.001 | 0.29 |
| age | -0.01 | 0.02 | -0.42 | .673 | 0.99 |  | -0.01 | 0.02 | -0.51 | .612 | 0.99 |
| Parenthood (1: not a parent, 2: parent) | 0.32 | 0.22 | 1.46 | .145 | 1.37 |  | 0.30 | 0.22 | 1.36 | .173 | 1.35 |
| dummy_self (0: present; 1: future) |  |  |  |  |  |  | -0.61 | 0.22 | -2.81 | .005 | 0.54 |
| Model fit | AIC = 520.76, R^2^ = .07 | | | | |  | AIC = 514.68, R^2^ = .09 | | | | |
|  |  | | | | |  | ΔAIC = -6.08, ΔR^2^ = .02, *p* = .004 | | | | |

*Note*. R^2^ indicates Cox and Snell R^2^.

Supplemental Appendix S1

*Instruction for the extended Social Value Orientation (SVO) task*

*Note*. These instructions were based on Van Lange, De Bruin, Otten, and Joireman^[1]^. Red-colored sentences indicate passages that differed between conditions.

**1. Present-self and present-other condition**

We would like you to participate in a virtual experiment. In this experiment, please imagine that you are paired with a randomly selected person. You have never seen your partner before, and will never see him/her again. In this experiment, we would like you and your partner to select one of four options (A, B, C, or D). Your decision will influence the number of points you and your partner will receive; similarly, your partner’s decision will also influence the number of points you and your partner will receive. Please imagine that the points have value. Thus, obtaining more points benefits you; more points also benefit your partner.

An example is presented below.

| Please select the most desirable of the four options below. | | | | |
| --- | --- | --- | --- | --- |
|  | A | B | C | D |
| Your points | 500 | 500 | 550 | 500 |
| Your partner’s points | 100 | 500 | 300 | 800 |

In this example, if you select option A, you will receive 500 points and your partner will receive 100 points. If you select option B, you will receive 500 points and your partner will also receive 500 points. If you select option C, you will receive 550 points and your partner will receive 300 points. If you select option D, you will receive 500 points and your partner will receive 800 points. In this manner, your decision reflects the number of points you would like yourself and your partner to receive.

Before commencing the experiment, we would like you to remember that there are no right or wrong answers regarding your choice. Please select the option that you find most desirable, regardless of the reason. Additionally, please remember that the points have value. Obtaining more points benefits you; similarly, obtaining more points also benefits your partner.

**2. Future-self and present-other condition**

We would like you to participate in a virtual experiment. In this experiment, please imagine that it is 40 years in the future, and that you are 40 years older than you are at present. Additionally, we would like you to imagine that you are paired with a randomly selected person from the present day. You have never seen your partner before, and will never see him/her again. In this experiment, we would like you and your partner to select one of four options (A, B, C, or D). Your decision will influence the number of points you and your partner will receive; similarly, your partner’s decision will also influence the number of points you and your partner will receive. Please imagine that the points have value. Thus, obtaining more points benefits you; more points also benefit your partner.

An example is presented below.

| Please select the most desirable of the four options below. | | | | |
| --- | --- | --- | --- | --- |
|  | A | B | C | D |
| Points you receive 40 years in the future | 500 | 500 | 550 | 500 |
| Points your partner receives in the present | 100 | 500 | 300 | 800 |

In this example, if you select option A, you will receive 500 points 40 years in the future and your partner will receive 100 points. If you select option B, you will receive 500 points 40 years in the future and your partner will receive 500 points. If you select option C, you will receive 550 points 40 years in the future and your partner will receive 300 points. If you select option D, you will receive 500 points 40 years in the future and your partner will receive 800 points. In this manner, your decision reflects the number of points you would like yourself and your partner to receive.

Before commencing the experiment, we would like you to remember that there are no right or wrong answers regarding your choice. Please select the option that you find most desirable, regardless of the reason. Additionally, please remember that the points have value. Obtaining more points benefits you; similarly, obtaining more points also benefits your partner.

**3. Present-self and future-other condition**

We would like you to participate in a virtual experiment. In this experiment, please imagine that you are paired with a randomly selected person who is living 40 years in the future. You have never seen your partner before, and will never see him/her again. In this experiment, we would like you and your partner to select one of four options (A, B, C, or D). Your decision will influence the number of points you and your partner will receive; similarly, your partner’s decision will also influence the number of points you and your partner will receive. Please imagine that the points have value. Thus, obtaining more points benefits you; more points also benefit your partner.

An example is presented below.

| Please select the most desirable of the four options below. | | | | |
| --- | --- | --- | --- | --- |
|  | A | B | C | D |
| Points you receive in the present | 500 | 500 | 550 | 500 |
| Points your partner will receive 40 years in the future | 100 | 500 | 300 | 800 |

In this example, if you select option A, you will receive 500 points and your partner, who lives 40 years in the future, will receive 100 points. If you select option B, you will receive 500 points and your partner, who lives 40 years in the future, will receive 500 points. If you select option C, you will receive 550 points and your partner, who lives 40 years in the future, will receive 300 points. If you select option D, you will receive 500 points and your partner, who lives 40 years in the future, will receive 800 points. In this manner, your decision reflects the number of points you would like yourself and your partner to receive.

Before commencing the experiment, we would like you to remember that there are no right or wrong answers regarding your choice. Please select the option that you find most desirable, regardless of the reason. Additionally, please remember that the points have value. Obtaining more points benefits you; similarly, obtaining more points also benefits your partner.

**4. Future-self and future-other condition**

We would like you to participate in a virtual experiment. In this experiment, please imagine that it is 40 years in the future, and that you are 40 years older than you are at present. Additionally, we would like you to imagine that you are paired with a randomly selected person who is living 40 years in the future. You have never seen your partner before, and will never see him/her again. In this experiment, we would like you and your partner to select one of four options (A, B, C, or D). Your decision will influence the number of points you and your partner will receive; similarly, your partner’s decision will also influence the number of points you and your partner will receive. Please imagine that the points have value. Thus, obtaining more points benefits you; more points also benefit your partner.

An example is presented below.

| Please select the most desirable of the four options below. | | | | |
| --- | --- | --- | --- | --- |
|  | A | B | C | D |
| Points you receive 40 years in the future | 500 | 500 | 550 | 500 |
| Points your partner will receive 40 years in the future | 100 | 500 | 300 | 800 |

In this example, if you select option A, you will receive 500 points 40 years in the future, and your partner, who lives 40 years in the future, will receive 100 points. If you select option B, you will receive 500 points 40 years in the future, and your partner, who lives 40 years in the future, will receive 500 points. If you select option C, you will receive 550 points 40 years in the future, and your partner, who lives 40 years in the future, will receive 300 points. If you select option D, you will receive 500 points 40 years the future, and your partner, who lives 40 years in the future, will receive 800 points. In this manner, your decision reflects the number of points you would like yourself and your partner to receive.

Before commencing the experiment, we would like you to remember that there are no right or wrong answers regarding your choice. Please select the option that you find most desirable, regardless of the reason. Additionally, please remember that the points have value. Obtaining more points benefits you; similarly, obtaining more points also benefits your partner.

Supplemental Appendix S2

*Distribution matrix in the extended SVO task*

*Note*. Distributions matrix were made according to Eek & Gärling^[2]^ .

| A | B | C | D |
| --- | --- | --- | --- |
| 520 | 460 | 460 | 460 |
| 260 | 460 | 60 | 760 |

| A | B | C | D |
| --- | --- | --- | --- |
| 470 | 470 | 470 | 530 |
| 470 | 770 | 70 | 270 |

| A | B | C | D |
| --- | --- | --- | --- |
| 520 | 520 | 520 | 580 |
| 120 | 820 | 520 | 320 |

| A | B | C | D |
| --- | --- | --- | --- |
| 550 | 610 | 550 | 550 |
| 550 | 350 | 150 | 850 |

| A | B | C | D |
| --- | --- | --- | --- |
| 540 | 600 | 540 | 540 |
| 840 | 340 | 540 | 140 |

| A | B | C | D |
| --- | --- | --- | --- |
| 540 | 480 | 480 | 480 |
| 280 | 480 | 780 | 80 |

| A | B | C | D |
| --- | --- | --- | --- |
| 620 | 560 | 560 | 560 |
| 360 | 160 | 860 | 560 |

| A | B | C | D |
| --- | --- | --- | --- |
| 510 | 510 | 570 | 510 |
| 810 | 510 | 310 | 110 |

| A | B | C | D |
| --- | --- | --- | --- |
| 490 | 490 | 550 | 490 |
| 490 | 90 | 290 | 790 |

| A | B | C | D |
| --- | --- | --- | --- |
| 450 | 450 | 510 | 450 |
| 750 | 50 | 250 | 450 |

| A | B | C | D |
| --- | --- | --- | --- |
| 500 | 560 | 500 | 500 |
| 100 | 300 | 800 | 500 |

| A | B | C | D |
| --- | --- | --- | --- |
| 530 | 530 | 530 | 590 |
| 130 | 830 | 530 | 330 |

Supplemental Appendix S3

*List of sample items for each scale*

*Note*. We exploratively examined ｓpersonality traits related to altruism toward contemporaries or concern for future others correlate to prosocial preferences toward future others. The scales and sample items are as follows.

| Scale | Sub-scale | Sample item |
| --- | --- | --- |
| Interpersonal Reactivity Index^[3][4]^ | personal distress | “In emergency situations, I feel apprehensive and ill-at-ease.” |
|  | empathic concern | “I often have tender, concerned feelings for people less fortunate than me.” |
|  | perspective taking | “Before criticizing somebody, I try to imagine how I would feel if I were in their place.” |
|  | fantasy scale | “I daydream and fantasize, with some regularity, about things that might happen to me.” |
| Consideration of Future Consequences^[5]^ |  | “Often I engage in a particular behavior in order to achieve outcomes that may not result for many years.” |
| Brief self-control scale^[6]^ |  | “I am good at resisting temptation.” |
| Generative Concern Scale^[7][8]^ | creating | “I feel that I am different from the majority of people.” |
|  | offering | “When I see a person who is feeling sad, I comfort them.” |
|  | maintaining | “I have made an effort to pass on knowledge I obtained through my experiences.” |
| Ten Item Personality Inventory^[9]^ | extraversion | “I see myself as extraverted, enthusiastic.” |
|  | agreeableness | “I see myself as sympathetic, warm.” |
|  | conscientiousness | “I see myself as dependable, self-disciplined.” |
|  | neuroticism | “I see myself as anxious, easily upset.” |
|  | openness | “I see myself as open to new experiences, complex.” |
|  |  |  |

Supplemental Appendix S4

*Descriptive statistic values for each variable, and results of analysis of variance*

|  |  |  | Present/Present | |  | Future/Present | |  | Present/Future | |  | Future/Future | |  |  |  |
| --- | --- | --- | --- | --- | --- | --- | --- | --- | --- | --- | --- | --- | --- | --- | --- | --- |
|  |  |  | (M: 82; W: 124) | |  | (M: 85; W: 121) | |  | (M: 73; W: 133) | |  | (M: 89; W: 117) | |  |  |  |
|  |  | α | *M* | *SD* |  | *M* | *SD* |  | *M* | *SD* |  | *M* | *SD* | *F* (3, 820) | *p* | η_p_^2^ |
|  | Age |  | 40.02 | 5.69 |  | 39.33 | 5.85 |  | 39.60 | 5.86 |  | 39.34 | 5.66 | 0.66 | .579 | < .01 |
| IRI | Personal Distress | .80 | 3.24 | 0.70 |  | 3.22 | 0.67 |  | 3.22 | 0.65 |  | 3.19 | 0.67 | 0.19 | .901 | < .01 |
| IRI | Empathic Concern | .79 | 3.49 | 0.59 |  | 3.42 | 0.56 |  | 3.42 | 0.65 |  | 3.32 | 0.58 | 2.86 | .036 | .01 |
| IRI | Perspective Taking | .69 | 3.05 | 0.52 |  | 3.06 | 0.56 |  | 3.07 | 0.53 |  | 3.04 | 0.59 | 0.09 | .968 | < .01 |
| IRI | Fantasy | .83 | 3.15 | 0.71 |  | 3.16 | 0.77 |  | 3.18 | 0.76 |  | 3.08 | 0.77 | 0.59 | .620 | < .01 |
|  | CFC | .74 | 3.20 | 0.45 |  | 3.17 | 0.43 |  | 3.23 | 0.47 |  | 3.14 | 0.47 | 1.40 | .242 | .01 |
|  | Self-Control | .84 | 3.03 | 0.55 |  | 2.99 | 0.61 |  | 3.01 | 0.59 |  | 2.97 | 0.60 | 0.40 | .756 | < .01 |
| GCS | Creating | .77 | 2.50 | 0.51 |  | 2.49 | 0.45 |  | 2.48 | 0.48 |  | 2.47 | 0.47 | 0.18 | .913 | < .01 |
| GCS | Offering | .78 | 2.59 | 0.52 |  | 2.59 | 0.47 |  | 2.60 | 0.49 |  | 2.49 | 0.50 | 2.32 | .074 | .01 |
| GCS | Maintaining | .82 | 2.03 | 0.62 |  | 1.98 | 0.56 |  | 2.04 | 0.60 |  | 1.99 | 0.58 | 0.50 | .684 | < .01 |
| TIPI | Extraversion | .48 | 3.54 | 1.48 |  | 3.60 | 1.34 |  | 3.68 | 1.35 |  | 3.41 | 1.30 | 1.42 | .234 | .01 |
| TIPI | Agreeableness | .26 | 4.84 | 1.03 |  | 4.81 | 1.11 |  | 4.78 | 1.15 |  | 4.55 | 1.24 | 2.72 | .044 | .01 |
| TIPI | Conscientiousness | .48 | 3.83 | 1.28 |  | 3.71 | 1.30 |  | 3.72 | 1.21 |  | 3.72 | 1.38 | 0.39 | .761 | < .01 |
| TIPI | Neuroticism | .42 | 4.37 | 1.31 |  | 4.41 | 1.19 |  | 4.40 | 1.39 |  | 4.41 | 1.26 | 0.04 | .991 | < .01 |
| TIPI | Openness | .40 | 3.78 | 1.30 |  | 3.66 | 1.24 |  | 3.72 | 1.27 |  | 3.63 | 1.24 | 0.59 | .620 | < .01 |

*Note*. Cronbach’s alphas for the Ten Item Personality Inventory indicate *the correlation coefficient (r*).

M: men; W: women; IRI: Interpersonal Reactivity Index; CFC: Consideration of Future Consequences; GCS: Generative Concern Scale; TIPI: Ten item Personality Inventory

Supplemental Appendix S5

*The relationship between psychological traits and SVO types in each condition*

*Note*. To investigate the relationship between psychological traits and SVO types in each condition, we conducted hierarchical logistic regression analysis. In step 1, we entered age and dummy variables for gender and parenthood (1 = not a parent, 2 = parent) as control variables. In step 2, we added all scales to the model as prediction variables. The variables were dummy variables for each SVO type. We conducted these logistic regression analyses in each condition; there were no significant results (Tables S9–S11).

Supplementary Table S9.

*Results of logistic regression analysis for proself dummy.*

|  |  | | Present/ Present | | | | |  | Future/ Present | | | | |  | Present/ Future | | | | |  | Future/ Future | | | | |
| --- | --- | --- | --- | --- | --- | --- | --- | --- | --- | --- | --- | --- | --- | --- | --- | --- | --- | --- | --- | --- | --- | --- | --- | --- | --- |
|  |  |  | Coef. | *SE* | *Z* | *p* | *OR* |  | Coef. | *SE* | *Z* | *p* | *OR* |  | Coef. | *SE* | *Z* | *p* | OR |  | Coef. | *SE* | *Z* | *p* | *OR* |
| step 1 |  | gender | 0.42 | 0.49 | 0.86 | .391 | 1.52 |  | -0.70 | 0.37 | -1.91 | .056 | 0.49 |  | -0.12 | 0.47 | -0.26 | .793 | 0.88 |  | -0.15 | 0.45 | -0.33 | .742 | 0.86 |
|  |  | age | 0.00 | 0.04 | -0.10 | .924 | 1.00 |  | 0.00 | 0.03 | -0.07 | .947 | 1.00 |  | 0.04 | 0.04 | 1.06 | .288 | 1.04 |  | -0.03 | 0.04 | -0.70 | .481 | 0.97 |
|  |  | parenthood | -0.67 | 0.45 | -1.47 | .143 | 0.51 |  | 0.04 | 0.36 | 0.13 | .900 | 1.05 |  | -0.38 | 0.46 | -0.82 | .410 | 0.68 |  | -0.46 | 0.45 | -1.02 | .308 | 0.63 |
| step 2 |  | gender | 0.41 | 0.57 | 0.72 | .473 | 1.51 |  | -1.24 | 0.49 | -2.52 | .012 | 0.29 |  | -0.32 | 0.55 | -0.58 | .562 | 0.73 |  | -0.34 | 0.53 | -0.64 | .525 | 0.71 |
|  |  | age | -0.01 | 0.05 | -0.24 | .812 | 0.99 |  | -0.01 | 0.04 | -0.16 | .876 | 0.99 |  | 0.05 | 0.05 | 1.06 | .288 | 1.05 |  | -0.03 | 0.04 | -0.65 | .517 | 0.97 |
|  |  | child | -0.53 | 0.53 | -1.00 | .317 | 0.59 |  | 0.15 | 0.42 | 0.35 | .730 | 1.16 |  | -0.27 | 0.53 | -0.50 | .615 | 0.77 |  | -0.91 | 0.55 | -1.65 | .099 | 0.40 |
|  | IRI | PD | 0.17 | 0.54 | 0.31 | .756 | 1.18 |  | 1.07 | 0.46 | 2.33 | .020 | 2.92 |  | 0.81 | 0.54 | 1.50 | .135 | 2.24 |  | -0.49 | 0.53 | -0.93 | .354 | 0.61 |
|  | IRI | EC | -0.78 | 0.61 | -1.28 | .199 | 0.46 |  | 0.13 | 0.54 | 0.24 | .807 | 1.14 |  | 0.78 | 0.59 | 1.32 | .188 | 2.18 |  | -0.66 | 0.54 | -1.22 | .224 | 0.52 |
|  | IRI | PT | -0.14 | 0.52 | -0.28 | .780 | 0.87 |  | -0.11 | 0.45 | -0.25 | .801 | 0.89 |  | -0.48 | 0.59 | -0.83 | .409 | 0.62 |  | 0.26 | 0.57 | 0.45 | .651 | 1.29 |
|  | IRI | FS | -0.07 | 0.41 | -0.17 | .865 | 0.93 |  | 0.05 | 0.31 | 0.14 | .885 | 1.05 |  | 0.10 | 0.38 | 0.27 | .788 | 1.11 |  | -0.12 | 0.37 | -0.34 | .735 | 0.88 |
|  |  | CFC | -0.90 | 0.59 | -1.53 | .126 | 0.41 |  | 0.21 | 0.46 | 0.45 | .652 | 1.23 |  | -0.17 | 0.59 | -0.29 | .772 | 0.84 |  | -0.22 | 0.62 | -0.35 | .725 | 0.80 |
|  |  | Self Regulation | 1.75 | 0.70 | 2.51 | .012 | 5.78 |  | 0.77 | 0.49 | 1.57 | .116 | 2.15 |  | 0.20 | 0.66 | 0.30 | .765 | 1.22 |  | 0.35 | 0.62 | 0.58 | .564 | 1.43 |
|  | GCS | Creativity | 1.37 | 0.76 | 1.81 | .071 | 3.92 |  | 0.38 | 0.61 | 0.63 | .527 | 1.47 |  | -0.12 | 0.71 | -0.18 | .861 | 0.88 |  | 0.97 | 0.70 | 1.38 | .167 | 2.65 |
|  | GCS | Offering | 0.44 | 0.79 | 0.55 | .579 | 1.55 |  | -0.41 | 0.72 | -0.57 | .566 | 0.66 |  | -1.18 | 0.85 | -1.39 | .164 | 0.31 |  | 1.95 | 0.90 | 2.17 | .030 | 7.03 |
|  | GCS | Maintaining | -0.57 | 0.52 | -1.11 | .268 | 0.56 |  | -0.55 | 0.46 | -1.18 | .237 | 0.58 |  | 0.15 | 0.48 | 0.32 | .752 | 1.16 |  | -0.59 | 0.62 | -0.96 | .339 | 0.56 |
|  | TIPI | Ext. | 0.09 | 0.21 | 0.40 | .687 | 1.09 |  | 0.09 | 0.18 | 0.47 | .639 | 1.09 |  | 0.07 | 0.20 | 0.36 | .717 | 1.08 |  | -0.23 | 0.23 | -1.01 | .314 | 0.79 |
|  | TIPI | Agr. | -0.07 | 0.28 | -0.26 | .793 | 0.93 |  | 0.18 | 0.21 | 0.86 | .391 | 1.19 |  | 0.18 | 0.27 | 0.69 | .487 | 1.20 |  | -0.11 | 0.24 | -0.47 | .642 | 0.89 |
|  | TIPI | Con. | -0.21 | 0.27 | -0.78 | .436 | 0.81 |  | -0.02 | 0.20 | -0.09 | .932 | 0.98 |  | -0.07 | 0.28 | -0.24 | .809 | 0.93 |  | -0.08 | 0.26 | -0.32 | .752 | 0.92 |
|  | TIPI | Neu. | 0.22 | 0.27 | 0.80 | .422 | 1.25 |  | -0.32 | 0.23 | -1.36 | .172 | 0.73 |  | -0.13 | 0.25 | -0.52 | .603 | 0.88 |  | -0.04 | 0.24 | -0.15 | .881 | 0.96 |
|  | TIPI | Ope. | -0.10 | 0.30 | -0.31 | .753 | 0.91 |  | -0.31 | 0.23 | -1.35 | .177 | 0.73 |  | 0.18 | 0.25 | 0.74 | .457 | 1.20 |  | -0.83 | 0.30 | -2.77 | .006 | 0.43 |
|  |  | step 1 | AIC = 153.55, R^2^ = .01 | | | | |  | AIC = 212.43, R^2^ = .02 | | | | |  | AIC = 145.96, R^2^ = .01 | | | | |  | AIC = 154.52, R^2^ = .01 | | | | |
|  |  | step 2 | AIC = 169.38, R^2^ = .07 | | | | |  | AIC = 225.96, R^2^ = .09 | | | | |  | AIC = 166.20, R^2^ = .05 | | | | |  | AIC = 167.12, R^2^ = .08 | | | | |
|  | model comparison | | ΔAIC = 15.83, ΔR^2^ = .06, *p* = .593 | | | | |  | ΔAIC = 13.53, ΔR^2^ = .07, *p* = .415 | | | | |  | ΔAIC = 20.24, ΔR^2^ = .04, *p* = .901 | | | | |  | ΔAIC = 12.60, ΔR^2^ = .07, *p* = .351 | | | | |

*Note*. Cronbach’s alphas of TIPI indicate correlation coefficient (*r*). R^2^ indicates Cox and Snell R^2^. gender: 1 = men, 2 = women; parenthood: 1 = not a parent, 2 = parent.

Supplementary Table S10.

*Results of logistic regression analysis for equality dummy.*

|  |  | | Present/ Present | | | | |  | Future/ Present | | | | |  | Present/ Future | | | | |  | Future/ Future | | | | |
| --- | --- | --- | --- | --- | --- | --- | --- | --- | --- | --- | --- | --- | --- | --- | --- | --- | --- | --- | --- | --- | --- | --- | --- | --- | --- |
|  |  |  | Coef. | *SE* | *Z* | *p* | *OR* |  | Coef. | *SE* | *Z* | *p* | OR |  | Coef. | *SE* | *Z* | *p* | *OR* |  | Coef. | *SE* | *Z* | *p* | *OR* |
| step 1 |  | gender | 0.91 | 0.34 | 2.65 | .008 | 2.48 |  | 0.51 | 0.31 | 1.62 | .104 | 1.67 |  | 1.82 | 0.36 | 5.03 | <.001 | 6.18 |  | 0.72 | 0.30 | 2.43 | .015 | 2.05 |
|  |  | age | 0.04 | 0.03 | 1.39 | .164 | 1.04 |  | 0.02 | 0.03 | 0.92 | .359 | 1.02 |  | 0.02 | 0.03 | 0.88 | .378 | 1.02 |  | 0.01 | 0.03 | 0.53 | .595 | 1.01 |
|  |  | parenthood | 0.43 | 0.33 | 1.32 | .187 | 1.54 |  | 0.11 | 0.30 | 0.37 | .714 | 1.12 |  | -0.48 | 0.32 | -1.52 | .129 | 0.62 |  | 0.02 | 0.29 | 0.06 | .956 | 1.02 |
| step 2 |  | gender | 0.98 | 0.41 | 2.36 | .018 | 2.66 |  | 0.69 | 0.40 | 1.73 | .083 | 2.00 |  | 1.76 | 0.41 | 4.32 | <.001 | 5.79 |  | 1.00 | 0.35 | 2.81 | .005 | 2.71 |
|  |  | age | 0.05 | 0.03 | 1.56 | .119 | 1.05 |  | 0.03 | 0.03 | 1.19 | .234 | 1.03 |  | 0.01 | 0.03 | 0.49 | .624 | 1.02 |  | 0.01 | 0.03 | 0.47 | .636 | 1.01 |
|  |  | child | 0.22 | 0.38 | 0.58 | .564 | 1.25 |  | 0.13 | 0.35 | 0.38 | .704 | 1.14 |  | -0.41 | 0.37 | -1.10 | .271 | 0.66 |  | 0.13 | 0.33 | 0.40 | .693 | 1.14 |
|  | IRI | PD | 0.02 | 0.38 | 0.04 | .966 | 1.02 |  | -0.56 | 0.37 | -1.52 | .129 | 0.57 |  | 0.18 | 0.34 | 0.52 | .601 | 1.20 |  | 0.23 | 0.35 | 0.65 | .513 | 1.25 |
|  | IRI | EC | 0.51 | 0.45 | 1.13 | .260 | 1.66 |  | -0.25 | 0.42 | -0.60 | .546 | 0.77 |  | -0.37 | 0.40 | -0.92 | .359 | 0.69 |  | 0.50 | 0.36 | 1.42 | .157 | 1.66 |
|  | IRI | PT | -0.20 | 0.39 | -0.51 | .608 | 0.82 |  | 0.03 | 0.37 | 0.09 | .929 | 1.03 |  | 0.71 | 0.42 | 1.68 | .092 | 2.03 |  | 0.28 | 0.36 | 0.78 | .438 | 1.32 |
|  | IRI | FS | 0.49 | 0.30 | 1.64 | .100 | 1.63 |  | 0.14 | 0.26 | 0.55 | .584 | 1.15 |  | -0.11 | 0.26 | -0.44 | .661 | 0.89 |  | -0.28 | 0.22 | -1.28 | .202 | 0.75 |
|  |  | CFC | -0.18 | 0.44 | -0.42 | .676 | 0.83 |  | -0.70 | 0.39 | -1.77 | .076 | 0.50 |  | 0.14 | 0.41 | 0.33 | .738 | 1.15 |  | 0.18 | 0.40 | 0.45 | .652 | 1.20 |
|  |  | Self Regulation | -0.71 | 0.49 | -1.44 | .151 | 0.49 |  | -0.49 | 0.39 | -1.25 | .212 | 0.61 |  | -0.16 | 0.44 | -0.37 | .713 | 0.85 |  | -0.17 | 0.40 | -0.43 | .667 | 0.84 |
|  | GCS | Creativity | -1.28 | 0.58 | -2.20 | .028 | 0.28 |  | -0.82 | 0.50 | -1.65 | .100 | 0.44 |  | -0.83 | 0.50 | -1.66 | .097 | 0.43 |  | -0.78 | 0.45 | -1.73 | .084 | 0.46 |
|  | GCS | Offering | -0.24 | 0.59 | -0.41 | .685 | 0.79 |  | 0.10 | 0.60 | 0.16 | .872 | 1.10 |  | 0.23 | 0.60 | 0.39 | .695 | 1.26 |  | -0.17 | 0.52 | -0.32 | .749 | 0.85 |
|  | GCS | Maintaining | 0.74 | 0.38 | 1.95 | .051 | 2.10 |  | 0.35 | 0.38 | 0.90 | .367 | 1.41 |  | -0.41 | 0.34 | -1.19 | .232 | 0.66 |  | 0.44 | 0.36 | 1.20 | .229 | 1.55 |
|  | TIPI | Ext. | -0.15 | 0.15 | -1.00 | .316 | 0.86 |  | 0.06 | 0.15 | 0.40 | .688 | 1.06 |  | 0.07 | 0.14 | 0.49 | .623 | 1.07 |  | -0.25 | 0.13 | -1.84 | .065 | 0.78 |
|  | TIPI | Agr. | 0.06 | 0.21 | 0.27 | .790 | 1.06 |  | 0.19 | 0.18 | 1.04 | .297 | 1.20 |  | 0.19 | 0.18 | 1.07 | .287 | 1.21 |  | -0.20 | 0.17 | -1.22 | .222 | 0.82 |
|  | TIPI | Con. | 0.22 | 0.20 | 1.11 | .266 | 1.25 |  | -0.12 | 0.16 | -0.72 | .472 | 0.89 |  | 0.00 | 0.20 | 0.02 | .983 | 1.00 |  | -0.20 | 0.17 | -1.20 | .230 | 0.82 |
|  | TIPI | Neu. | -0.11 | 0.20 | -0.54 | .590 | 0.90 |  | 0.15 | 0.19 | 0.78 | .434 | 1.16 |  | -0.04 | 0.16 | -0.26 | .793 | 0.96 |  | -0.27 | 0.16 | -1.66 | .097 | 0.76 |
|  | TIPI | Ope. | 0.13 | 0.23 | 0.57 | .569 | 1.14 |  | 0.18 | 0.19 | 0.92 | .356 | 1.20 |  | 0.28 | 0.17 | 1.63 | .103 | 1.32 |  | 0.29 | 0.17 | 1.71 | .087 | 1.34 |
|  |  | step 1 | AIC = 240.17, R^2^ = .05 | | | | |  | AIC = 275.73, R^2^ = .02 | | | | |  | AIC = 259.04, R^2^ = .14 | | | | |  | AIC = 286.54, R^2^ = .03 | | | | |
|  |  | step 2 | AIC = 252.46, R^2^ = .12 | | | | |  | AIC = 289.41, R^2^ = .08 | | | | |  | AIC = 275.55, R^2^ = .18 | | | | |  | AIC = 295.79, R^2^ = .11 | | | | |
|  | model comparison | | ΔAIC = 12.29, ΔR^2^ = .07, *p* = .331 | | | | |  | ΔAIC = 13.68, ΔR^2^ = .07, *p* = .426 | | | | |  | ΔAIC = 16.51, ΔR^2^ = .05, *p* = .648 | | | | |  | ΔAIC = 9.25, ΔR^2^ = .08, *p* = .175 | | | | |

*Note*. Cronbach’s alphas of TIPI indicate correlation coefficient (*r*). R^2^ indicates Cox and Snell R^2^. gender: 1 = men, 2 = women; parenthood: 1 = not a parent, 2 = parent.

Supplementary Table S11.

*Results of logistic regression analysis for joint outcome dummy.*

|  | | | Present/ Present | | | | |  | Future/ Present | | | | |  | Present/ Future | | | | | |  | | Future/ Future | | | | | |
| --- | --- | --- | --- | --- | --- | --- | --- | --- | --- | --- | --- | --- | --- | --- | --- | --- | --- | --- | --- | --- | --- | --- | --- | --- | --- | --- | --- | --- |
|  |  |  | Coef. | *SE* | *Z* | *p* | *OR* |  | Coef. | *SE* | *Z* | *p* | *OR* |  | Coef. | *SE* | *Z* | *p* | OR | |  | | Coef. | *SE* | *Z* | *p* | *OR* | |
| step 1 |  | gender | -2.29 | 0.56 | -4.11 | <.001 | 0.10 |  | -0.36 | 0.31 | -1.15 | .249 | 0.70 |  | -1.50 | 0.33 | -4.60 | <.001 | 0.22 | |  | | -0.95 | 0.32 | -2.97 | .003 | 0.39 | |
|  |  | age | -0.07 | 0.04 | -1.59 | .111 | 0.94 |  | -0.02 | 0.03 | -0.95 | .342 | 0.98 |  | -0.02 | 0.03 | -0.89 | .374 | 0.98 | |  | | 0.01 | 0.03 | 0.29 | .773 | 1.01 | |
|  |  | parenthood | -0.45 | 0.47 | -0.96 | .339 | 0.64 |  | -0.04 | 0.30 | -0.15 | .882 | 0.96 |  | 0.50 | 0.31 | 1.61 | .108 | 1.66 | |  | | 0.11 | 0.31 | 0.35 | .724 | 1.12 | |
| step 2 |  | gender | -2.51 | 0.67 | -3.74 | .000 | 0.08 |  | -0.48 | 0.40 | -1.22 | .224 | 0.62 |  | -1.41 | 0.37 | -3.83 | <.001 | 0.24 | |  | | -1.15 | 0.38 | -3.01 | .003 | 0.32 | |
|  |  | age | -0.07 | 0.05 | -1.54 | .123 | 0.93 |  | -0.03 | 0.03 | -1.19 | .232 | 0.97 |  | -0.02 | 0.03 | -0.69 | .493 | 0.98 | |  | | 0.00 | 0.03 | 0.11 | .911 | 1.00 | |
|  |  | child | -0.34 | 0.57 | -0.59 | .558 | 0.71 |  | -0.12 | 0.35 | -0.34 | .737 | 0.89 |  | 0.34 | 0.36 | 0.96 | .339 | 1.41 | |  | | 0.01 | 0.36 | 0.03 | .977 | 1.01 | |
|  |  | PD | 0.12 | 0.56 | 0.22 | .828 | 1.13 |  | -0.25 | 0.36 | -0.69 | .488 | 0.78 |  | -0.48 | 0.34 | -1.41 | .159 | 0.62 | |  | | 0.11 | 0.37 | 0.29 | .770 | 1.11 | |
|  |  | EC | -0.99 | 0.68 | -1.46 | .145 | 0.37 |  | 0.54 | 0.43 | 1.26 | .208 | 1.72 |  | 0.26 | 0.38 | 0.69 | .492 | 1.30 | |  | | -0.08 | 0.39 | -0.19 | .846 | 0.93 | |
|  |  | PT | 0.87 | 0.61 | 1.42 | .155 | 2.38 |  | 0.11 | 0.37 | 0.30 | .767 | 1.12 |  | -0.49 | 0.39 | -1.24 | .214 | 0.61 | |  | | -0.37 | 0.39 | -0.94 | .345 | 0.69 | |
|  | IRI | FS | -0.71 | 0.42 | -1.68 | .094 | 0.49 |  | -0.07 | 0.26 | -0.27 | .784 | 0.93 |  | 0.01 | 0.25 | 0.03 | .973 | 1.01 | |  | | 0.24 | 0.24 | 0.98 | .328 | 1.27 | |
|  | IRI | CFC | 1.03 | 0.66 | 1.56 | .118 | 2.80 |  | 0.11 | 0.39 | 0.29 | .772 | 1.12 |  | 0.06 | 0.38 | 0.16 | .875 | 1.06 | |  | | 0.14 | 0.43 | 0.34 | .737 | 1.15 | |
|  | IRI | Self Regulation | -0.54 | 0.73 | -0.75 | .455 | 0.58 |  | 0.34 | 0.40 | 0.87 | .385 | 1.41 |  | 0.08 | 0.41 | 0.18 | .855 | 1.08 | |  | | 0.14 | 0.45 | 0.32 | .749 | 1.15 | |
|  | IRI | Creativity | 0.41 | 0.80 | 0.51 | .613 | 1.50 |  | 0.23 | 0.49 | 0.48 | .631 | 1.26 |  | 0.37 | 0.47 | 0.79 | .430 | 1.45 | |  | | 0.46 | 0.48 | 0.94 | .345 | 1.58 | |
|  |  | Offering | 0.98 | 0.91 | 1.08 | .282 | 2.67 |  | 0.19 | 0.60 | 0.32 | .752 | 1.21 |  | 0.20 | 0.55 | 0.35 | .725 | 1.22 | |  | | -0.63 | 0.56 | -1.14 | .256 | 0.53 | |
|  |  | Maintaining | -0.90 | 0.55 | -1.63 | .103 | 0.41 |  | -0.19 | 0.37 | -0.51 | .611 | 0.83 |  | 0.16 | 0.33 | 0.47 | .637 | 1.17 | |  | | -0.17 | 0.38 | -0.43 | .666 | 0.85 | |
|  | GCS | Extraversion | 0.07 | 0.21 | 0.31 | .757 | 1.07 |  | -0.12 | 0.15 | -0.78 | .435 | 0.89 |  | -0.02 | 0.13 | -0.14 | .889 | 0.98 | |  | | 0.21 | 0.14 | 1.52 | .128 | 1.24 | |
|  | GCS | Agr. | 0.02 | 0.30 | 0.06 | .950 | 1.02 |  | -0.26 | 0.18 | -1.49 | .136 | 0.77 |  | -0.20 | 0.18 | -1.12 | .264 | 0.82 | |  | | 0.40 | 0.18 | 2.20 | .028 | 1.49 | |
|  | GCS | Con. | -0.17 | 0.30 | -0.58 | .561 | 0.84 |  | 0.14 | 0.16 | 0.85 | .395 | 1.15 |  | -0.01 | 0.19 | -0.04 | .965 | 0.99 | |  | | 0.30 | 0.18 | 1.63 | .104 | 1.34 | |
|  | TIPI | Neu. | 0.02 | 0.30 | 0.05 | .958 | 1.02 |  | 0.03 | 0.18 | 0.14 | .890 | 1.03 |  | 0.10 | 0.16 | 0.61 | .543 | 1.10 | |  | | 0.23 | 0.17 | 1.33 | .183 | 1.25 | |
|  | TIPI | Ope. | 0.15 | 0.33 | 0.46 | .646 | 1.16 |  | -0.09 | 0.19 | -0.44 | .659 | 0.92 |  | -0.30 | 0.17 | -1.79 | .073 | 0.74 | |  | | -0.07 | 0.18 | -0.40 | .692 | 0.93 | |
|  |  | step 1 | AIC = 139.53, R^2^ = .11 | | | | |  | AIC = 275.03, R^2^ = .01 | | | | |  | AIC = 265.26, R^2^ = .11 | | | | |  | | AIC = 253.20, R^2^ = .05 | | | | | |  |
|  |  | step 2 | AIC = 154.27, R^2^ = .17 | | | | |  | AIC = 292.21, R^2^ = .06 | | | | |  | AIC = 285.16, R^2^ = .14 | | | | |  | | AIC = 265.94, R^2^ = .12 | | | | | |  |
|  | model comparison | | ΔAIC = 14.74, ΔR^2^ = .06, *p* = .507 | | | | |  | ΔAIC = 17.18, ΔR^2^ = .05, *p* = .700 | | | | |  | ΔAIC = 19.90, ΔR^2^ = .03, *p* = .884 | | | | |  | | ΔAIC = 12.74, ΔR^2^ = .07, *p* = .361 | | | | | |  |

*Note*. Cronbach’s alphas of TIPI indicate correlation coefficient (*r*). R^2^ indicates Cox and Snell R^2^. gender: 1 = men, 2 = women; parenthood: 1 = not a parent, 2 = parent.

**References**

1. Van Lange, P. A. M., De Bruin, E. M. N., Otten, W. & Joireman, J. A. Development of prosocial, individualistic, and competitive orientations: Theory and preliminary evidence. *J. Pers. Soc. Psychol.* **73**, 733-746 (1997).

2. Eek, D. & Gärling, T. Prosocials prefer equal outcomes to maximizing joint outcomes. *Br. J. Soc. Psychol.* **45**, 321-337 (2006).

3. Davis, M. H. A multidimensional approach to individual differences in empathy. *JSAS Cat. Sel. Doc. Psychol.* **10** (1980).

4. Davis, M. H. Measuring individual differences in empathy: Evidence for a multidimensional approach. *J. Pers. Soc. Psychol.* **44**, 113-126 (1983).

5. Strathman, A., Gleicher, F., Boninger, D. S. & Edwards, C. S. The consideration of future consequences: Weighing immediate and distant outcomes of behavior. *J. Pers. Soc. Psychol.* **66**, 742-752 (1994).

6. Tangney, J. P., Baumeister, R. F. & Boone, A. L. High self-control predicts good adjustment, less pathology, better grades, and interpersonal success. *J. Pers.* **72**, 271-324 (2004).

7. McAdams, D. P. & de St.Aubin, E. A theory of generativity and its assessment through self-report, behavioral acts, and narrative themes in autobiography. *J. Pers. Soc. Psychol.* **62**, 1003-1015 (1992).

8. Marushima, R. & Arimitsu, K. Revised generative concern scale and generative behavior checklist (GCS-R, GBC-R): scale reconstruction, reliability, and validity. *Jpn. J. Psychol.* **78**, 303-309 (2007).

9. Gosling, S. D., Rentfrow, P. J. & Swann, W. B. A very brief measure of the Big-Five personality domains. *J. Res. Personal.* **37**, 504-528 (2003).
